# Exploring illness uncertainty categories in ischemic stroke patients and the relationship with perceived social support: a latent class analysis

**DOI:** 10.3389/fpsyg.2025.1578691

**Published:** 2025-05-27

**Authors:** Xin Wen, Min Shi, Jie Zhou, Wanfang Yuan, Rong Tang, Ruhui Xu, Wenyi Zhu

**Affiliations:** Department of Neurology, The Second People’s Hospital of Yibin, Yibin, China

**Keywords:** illness uncertainty, perceived social support, latent class analysis, nursing, ischemic stroke

## Abstract

**Objectives:**

This study examined the categories of illness uncertainty and their influencing factors in ischemic stroke patients and analyzed their relationship with perceived social support.

**Methods:**

Patients with ischemic stroke who were admitted to the neurology department of a tertiary general hospital in Yibin City from June to December 2024 were selected for this study. The general information questionnaire, the Mishel Uncertainty in illness scale, and the perceived social support scale were used. Latent class analysis was carried out based on the patients’ illness uncertainty and explored the relationship with perceived social support.

**Results:**

Illness uncertainty in ischemic stroke patients could be classified into 3 different latent classes, namely, “low-level-unpredictability group” (16.43%), “medium-level group” (14.52%), and “high-level-complexity group” (69.05%). Logistic regression analysis showed that place of residency, educational level, per capita monthly household income, number of comorbid other chronic diseases, mRS scores, and self-care ability were the factors influencing the latent class of illness uncertainty in stroke patients (*p* < 0.05). The difference between the three latent classes of illness uncertainty in ischemic stroke patients was statistically significant in the perceived social support score (*p* < 0.05).

**Conclusion:**

Illness uncertainty in ischemic stroke patients has a distinct categorical class and perceived social support differs for each category. Targeted interventions should be carried out according to the categorization of patients’ illness uncertainty class traits in order to reduce the level of patients’ illness uncertainty and promote physical and mental health.

## 1 Introduction

A related guideline published by the American Stroke Association in 2024 mentions that 600,000 residents of the United States experience a stroke each year, making it one of the leading causes of adult disability (Bushnell et al., 2024). Ischemic stroke is the most common type of stroke, and studies have shown that ischemic stroke accounts for 62.4% of new strokes. It is characterized by high morbidity, disability and recurrence rates, placing a heavy burden on families and society, especially in developing countries and aging societies.

Illness uncertainty is a cognitive experience resulting from the patient’s inability to predict and judge the outcome of something related to the disease (Mishel, 1988). Studies have shown that patients with ischemic stroke have a moderate level of illness uncertainty (Tan, 2023). Chunping’s team found that ischemic stroke patients have more uncertainty, especially those with poorer functional status, other chronic diseases, and longer stroke duration (Ni et al., 2018). Patients are prone to a sense of loss of control over the disease and consequently a strong illness uncertainty due to a lack of knowledge about the disease and uncertainty about the prognosis. The study finds that (Xu, 2018) illness uncertainty affects the communication between them and medical personnel, which is not conducive to the treatment and recovery of the disease. In addition, it can interrupt patients’ disease treatment and affect the disease prognosis. Previous research has shown that perceived social support can be effective in reducing an individual’s illness uncertainty and is an important external resource (Zheng et al., 2024). Most of the previous studies on illness uncertainty in ischemic stroke patients have relied solely on scale total scores to classify illness uncertainty, ignoring the internal variability among patient groups. A phenomenological interview with ICU patients and families mentioned “not knowing if they are getting better” as a specific theme of the study, i.e., uncertainty about the prognosis of the disease was the main source of uncertainty about the illness for ICU patients and families (Cypress, 2016). A study of rheumatoid arthritis patients by Cleanthous et al. indicated that the uncertainty experienced by patients stemmed primarily from a lack of full understanding of the symptoms (Cleanthous et al., 2013). Thus, illness uncertainty is heterogeneous across groups, and whether it is similarly heterogeneous in patients with ischemic stroke remains to be investigated. Latent Class Analysis (LPA) is an emerging individual-centered statistical analysis method that classifies research subjects based on their latent characteristics. Currently, this method has been applied in many fields, such as psychology, education, sociology, and so on (Wang, 2014). Therefore, this study used latent class analysis to identify potential categories of illness uncertainty in ischemic stroke patients and further explored the relationship between different classes of illness uncertainty and perceived social support. In order to develop relevant interventions for identification and prevention based on different potential subtypes of illness uncertainty, to reduce illness uncertainty, and improve quality of life.

## 2 Methods

### 2.1 Study design and participants

**Figure 1:**
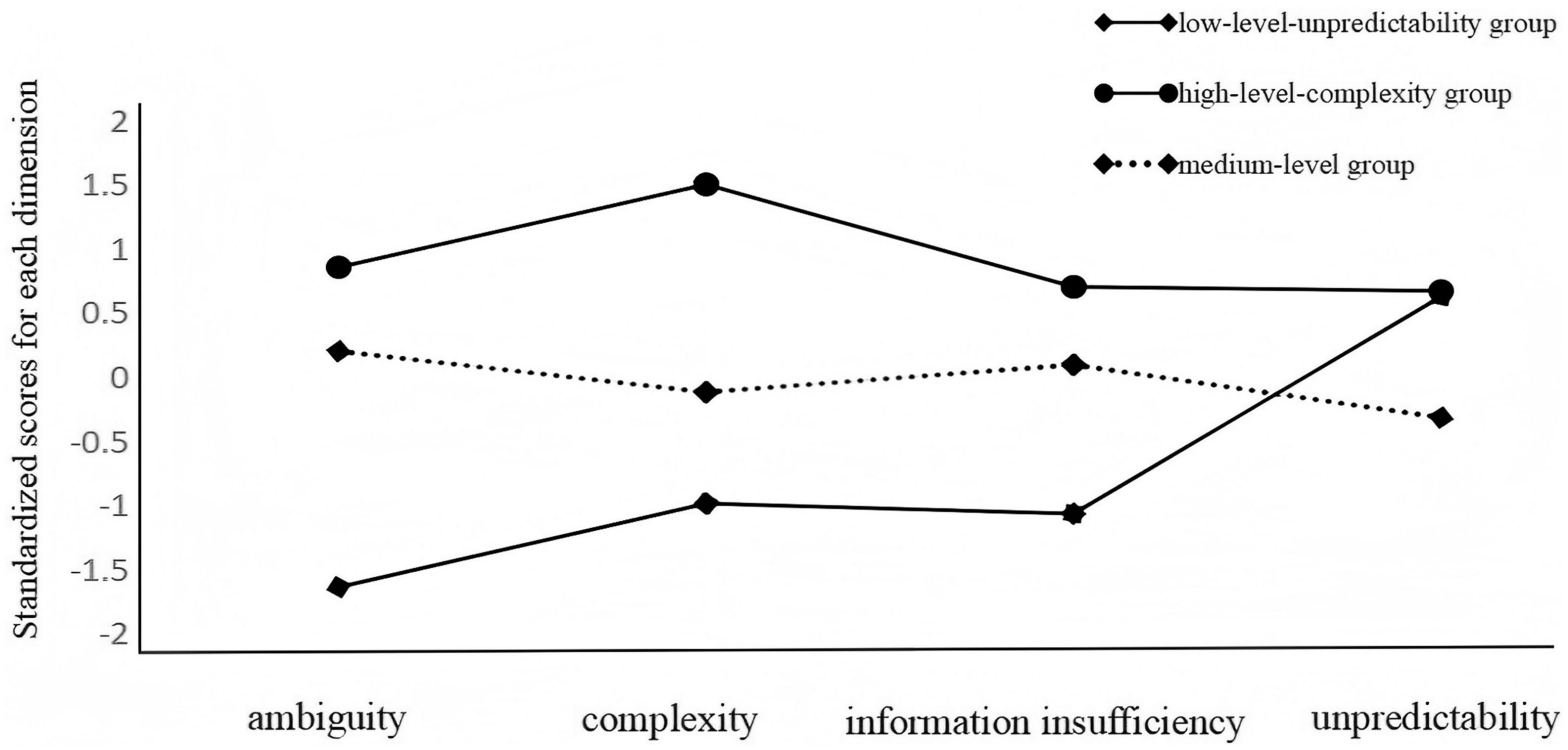
Distribution of characteristics of potential category groups for illness uncertainty.

This is a cross-sectional, observational study using a convenience sampling method. The study was conducted from June to December 2024 in a tertiary general hospital in Yibin, China, among patients diagnosed with ischemic stroke. Inclusion criteria for patients: (1) met the Chinese diagnostic criteria for stroke and was diagnosed with ischaemic stroke by imaging (CT or MRI) (Chinese Medical Association, Neurology Section, and Cerebrovascular Disease Group of the Neurology Branch of the Chinese Medical Association, 2018); (2) was over 18 years old; (3) was conscious and had stable vital signs; and (4) signed an informed consent form. Exclusion criteria: (1) patients with mental illness and cognitive impairment; (2) patients with speech disorders or hearing loss disorders due to illness or other reasons. In this study, Sample size was determined according to Peduzzi et al. (1996) sample calculation equation: sample numbers = max (dimension number*10)*[1 + (10%–20%)]. A total of 17 variables were included in this study (10 dimensions of the General Information Questionnaire, 4 dimensions of the Mishel Uncertainty in Illness Scale, and 3 dimensions of the Perceived Social Support Scale), and taking into account 20% of invalid questionnaires, a sample size of at least 204 was calculated. Finally, 420 ischemic stroke patients were included in this study (Figure 1).

### 2.2 Instrument

#### 2.2.1 General information questionnaire

There are two parts: general demographic information and disease-related information. General demographic information includes: age, gender, place of residency, marital status, educational level, per capita monthly household income, and payment of medical expenses. Disease-related information includes: types of stroke onset, number of comorbid other chronic diseases, self-care ability and mRS (Modified Rankin Scale) score.

#### 2.2.2 Mishel uncertainty in illness scale

The Mishel Uncertainty in Illness Scale (MUIS) was developed by Mishel (1988), an American nursing scholar, and the Chinese version was translated by Xu and Huang (1996). The scale contains 33 items in four dimensions, including ambiguity complexity, unpredictability and information insufficiency, with the 15th item not counting toward the total score. Each item is rated on a Likert 5 scale from 1 to 5. Higher scores indicate higher levels of illness uncertainty. The scale was applied to the Chinese patient population (Zhang et al., 2022); the Cronbach’s *α* of this study is 0.803.

#### 2.2.3 Perceived social support scale

The Perceived Social Support Scale (PSSS), developed by Zimet et al. (1990) and translated into Chinese by Jiang et al. (1996) measures the degree of social support perceived by individuals. There are 3 dimensions (family support, friend support, and other support) with 12 items. The scale is rated on a 7-point Likert scale, with scores positively related to perceived social support. The scale was applied to the Chinese patient population (Wang et al., 2022), the Cronbach’s *α* of this study is 0.877.

### 2.3 Data collection

The study was conducted in the inpatient neurology ward of the hospital using a face-to-face approach. Uniform training was provided to the investigators before conducting the survey. Investigators screened patients who met the inclusion and exclusion criteria, explained the purpose, methodology, and precautions for completing the survey before distributing the questionnaires, and distributed the questionnaires after obtaining the patients’ consent and signing the informed consent form. If the patient is not able to fill in the questionnaire due to other reasons, etc., the investigator should assist him/her to complete the questionnaire. The questionnaires were collected on the spot after completion.

### 2.4 Data analysis

Excel was used to double-enter and save the data from the profile, and SPSS Statistics 26.0 and Mplus 8.0 were used to statistically analyze the data. The scores on the illness uncertainty scale were standardized with a z-score in order to observe and name the differences between the different types. A latent class model for analyzing illness uncertainty in ischemic stroke patients using Mplus 8.0. Model fitting was performed starting with an initial model (assuming the existence of one potential category), and then the number of potential categories was sequentially increased to test the fitness of each model and select the best potential category model. The model fitting metrics include Akaike information criterion (AIC), Bayesian information criterion (BIC), adjusted Bayesian (aBIC), the entropy, LoMendellRubin calibrated likelihood ratio (Lo–Mendell–Rubin adjusted likelihood ratio test, LMRT), and bootstrap-based likelihood ratio test index (BLRT). Among them, the smaller AIC, BIC, and aBIC are, the better the model fit; the entropy is between 0 and 1, and the closer it is to 1, the more accurate the classification is; LMRT and BLRT are used to compare the fitting differences between k-category and k-1-category models, and the difference is statistically significant (*p* < 0.05) when it indicates that the k-category profile model is better than the k-1-category profile model. After determining the best latent class model, statistical analysis was performed using SPSS 26.0 software. Measurements were described using means, standard deviations, or medians and quartiles, and counts were described using frequencies and percentages. Between-group comparisons were made using the identified latent class as a grouping variable, and impact factor analyses were performed using multivariate logistic regression, with *p* < 0.05 indicating statistically significant differences.

### 2.5 Ethical considerations

The study was approved by the Ethics Committee of the Second People’s Hospital of Yibin with approval no. 2024–229-01.

## 3 Results

### 3.1 Participant demographics

This study included 420 patients with ischemic stroke. Age was (65.33 ± 11.40) years; 288 (68.6%) were male and 132 (31.4%) were female; education level was elementary school and below in 285 cases (67.9%); 377 (89.8%) were married patients; residence was in the rural area in 250 cases (59.5%) and the urban area in 170 cases (40.5%); first-ever-onset patients were 316 cases (75.2%); mRS scores of 4 and above in 129 cases (30.7%); the number of comorbid other chronic diseases was 3 and above in 105 cases (25.0%); and most of the self-care ability was no need for dependence (32.9%) and mild dependence (33.3%).

### 3.2 Latent class model of illness uncertainty

In this study, latent classes were analyzed based on the 4 dimensions of the illness uncertainty scale. Model 1 was used as the initial model, and the number of classes in the model was gradually increased to fit a total of 1–5 types of latent class models. In models 1 to 5, the values of AIC, BIC, and aBIC decreased as the number of classes increased. The pLMR of model 5 was >0.05. The fitting index of model 4 was optimal, but the proportion of one class was just at the minimum criterion of 5%, which was at the critical value, indicating that the sample size of this class was small, which might lead to the instability of the model parameters. The comprehensive analysis finally classified the illness uncertainty of ischemic stroke patients into three latent classes. The attribution probabilities of the three latent classes of illness uncertainty were 89.6, 89.9, and 92.5%, respectively, which were all >80%, indicating that the latent class model is reliable (see Table 1).

**Table 1:** Model fit indices of different latent class models.

Model	k	AIC	BIC	aBIC	Entropy	LMR	BLRT	Conditional probability
1	8	4779.629	4811.951	4786.564				1.000
2	13	4531.471	4583.994	4542.741	0.824	<0.001	<0.001	0.217/0.783
3	18	4414.078	4486.802	4429.683	0.816	<0.001	<0.001	0.164/0.690/0.145
4	23	4337.731	4430.657	4357.671	0.832	0.0139	<0.001	0.188/0.054/0.579/0.179
5	28	4235.223	4348.350	4259.497	0.865	0.0503	<0.001	0.055/0.217/0.221/0.064/0.443

AIC, Akaike information criterion; BIC, Bayesian information criterion; aBIC, adjusted Bayesian information criterion; LMR, Lo–Mendell–Rubin adjusted likelihood ratio test; BLRT, bootstrapped likelihood ratio test.

Patients in Class 1 had a low total illness uncertainty score of (68.87 ± 8.87) and scored highest on the unpredictability dimension, hence, this class was named “low-level-unpredictability group” with 69 patients (16.43%). The total illness uncertainty score for Class 2 was moderate (88.62 ± 7.10), with a relatively even score on all dimensions, and therefore this category was named the “medium-level group,” with a total of 290 patients (69.05%). The total illness uncertainty score for Class 3 was a high level (106.67 ± 6.05), and scored high on the complexity dimension, so this category was named the “high-level-complexity group” with 61 patients (14.52%).

### 3.3 Univariate analysis of a latent class of illness uncertainty in ischemic stroke patients

After determining the best latent class model, statistical analyses were performed using SPSS 26.0 software, and categorical information was analyzed using the chi-square test or Fisher’s exact probability method. The results showed statistically significant differences in place of residency, educational level, per capita monthly household income, number of comorbid other chronic diseases, self-care ability, and mRS scores in the comparison of the three latent classes of illness uncertainty in ischemic stroke patients (*p* < 0.05) (see Table 2).

**Table 2:** Comparison of general information on patients in three latent illness uncertainty classes.

Variables	Group			*χ* ^2^	*P*
	Class1 (*n* = 69)	Class 2 (*n* = 290)	Class 3 (*n* = 61)		
Gender					
Men	48 (69.6)	203 (70)	37 (60.7)	2.080	0.353
Women	21 (30.4)	87 (30)	24 (39.3)		
Educational level					
Elementary school or below	35 (50.7)	209 (72.1)	41 (67.2)	33.038	<0.001**
Junior high school	15 (21.7)	53 (18.3)	11 (18.1)		
Senior high school	10 (14.5)	25 (8.6)	8 (13.1)		
College or above	9 (13.0)	3 (1.0)	1 (1.6)		
Marital status					
Unmarried	3 (4.3)	1 (0.3)	0 (0)	12.087	0.060
Married	61 (88.4)	261 (90.0)	55 (90.2)		
Divorced	1 (1.4)	6 (2.1)	0 (0)		
Widowed	4 (5.8)	22 (7.6)	6 (9.8)		
Place of residency					
Rural	27 (39.1)	179 (61.7)	44 (72.1)	16.518	<0.001**
Urban	42 (60.9)	111 (38.3)	17 (14.5)		
Per capita monthly household income					
≦1,000 CNY	3 (4.3)	67 (23.1)	20 (32.8)	34.690	<0.001**
1,001–3000CNY	17 (24.6)	87 (30.0)	19 (31.1)		
3,001–5000CNY	16 (23.2)	78 (26.9)	11 (18.0)		
>5000CNY	33 (47.8)	58 (20.0)	11 (18.0)		
Payment of medical expenses					
Self-finance	0 (0)	14 (4.8)	5 (8.2)	6.395	0.172
Employee medical insurance	21 (30.4)	72 (24.8)	18 (29.5)		
Resident medical insurance	48 (69.6)	204 (70.3)	38 (62.3)		
Types of stroke onset					
First episode	51 (73.9)	218 (75.2)	47 (77.0)	0.173	0.917
Re-episode	18 (17.3)	72 (24.8)	14 (23.0)		
Number of comorbid other chronic diseases					
0	19 (27.5)	45 (15.5)	7 (11.5)	18.824	0.004**
1	23 (33.3)	79 (27.2)	17 (27.9)		
2	19 (27.5)	93 (32.1)	13 (21.3)		
3 and above	8 (11.6)	73 (25.2)	24 (39.3)		
Self-care ability					
No dependency required	30 (43.5)	95 (32.8)	13 (21.3)	14.993	0.020*
Mildly dependent	25 (36.2)	97 (33.4)	18 (29.5)		
Moderately dependent	9 (13.0)	51 (17.6)	13 (21.3)		
Heavily dependent	5 (7.2)	47 (16.2)	17 (27.9)		
mRS score					
1 point and below	26 (37.7)	78 (26.9)	21 (34.4)	14.838	0.022*
2 points	5 (7.2)	61 (21.0)	5 (8.2)		
3 points	18 (26.1)	66 (22.8)	11 (18.0)		
4 points and above	20 (29.0)	85 (29.3)	24 (39.3)		

MRS, Modified Rankin Scale; Class 1, low-level-unpredictability group; Class 2, medium-level group; Class 3, high-level-complexity group. *p < 0.05, **p < 0.01.

### 3.4 Logistic regression analysis of latent classes of illness uncertainty in ischemic stroke patients

The latent class of illness uncertainty in ischemic stroke patients was used as the dependent variable (the low-level-unpredictability group was used as a reference). Variables that were statistically significant in univariate analyses were used as independent variables. For multivariate analysis, an unordered multicategorical logistic regression model was developed (Table 3).

**Table 3:** Logistic regression analysis of three latent illness uncertainty classes.

Variables	*β*	SE	Wald *χ*^2^	*P*	OR	95% CI
Class 3 (cf. Class 1)						
Place of residency						
Rural	1.34	0.451	8.829	0.003	3.818	1.578–9.24
Per capita monthly household income (cf.>5,000 CNY)						
≦1,000 CNY	2.522	0.768	10.784	0.001	12.459	2.765–56.145
Number of comorbidities of other chronic diseases (cf.3 and above)						
0	−2.143	0.686	9.771	0.002	0.117	0.031–0.45
1	−1.633	0.588	7.714	0.005	0.195	0.062–0.618
2	−1.603	0.602	7.087	0.008	0.201	0.062–0.655
Self-care ability (cf. Heavily dependent)						
No dependency required	−2.158	0.733	8.654	0.003	0.116	0.027–0.487
Mildly dependent	−1.747	0.702	6.187	0.013	0.174	0.044–0.691
mRS score (cf.4 points and above)						
1 point and below	1.191	0.553	4.642	0.031	3.291	1.114–9.724
Class 2 (cf. Class 1)						
Educational level (cf. College and above)						
Elementary school and below	2.19	0.797	7.555	0.006	8.935	1.875–42.587
Junior high school	1.804	0.791	5.202	0.023	6.076	1.289–28.643
Senior high school	1.718	0.83	4.287	0.038	0.038	1.096–28.33
Per capita monthly household income (cf. >5,000 CNY)						
≦1,000 CNY	2.255	0.672	11.254	0.001	9.537	2.554–35.614
Number of comorbidities of other chronic diseases (cf. 3 and above)						
0	−1.495	0.536	7.774	0.005	0.224	0.078–0.641
1	−1.349	0.498	7.326	0.007	0.259	0.098–0.689
Self-care ability (cf. Heavily dependent)						
No dependency required	−1.227	0.602	4.156	0.041	0.293	0.09–0.954
mRS score (cf. 4 points and above)						
2 points	1.872	0.594	9.94	0.002	6.5	2.03–20.812

LR, likelihood ratio = 108.207, *P* < 0.001; Class 1, low-level-unpredictability group; Class 2, medium-level group; Class 3, high-level-complexity group.

The results showed that when comparing the “high-level-complexity group” with the “low-level-unpredictability group,” patients with rural residence (*OR* = 3.818, *p* = 0.003), per capita monthly household income of <1,000 CNY (*OR* = 12.459*, p* = 0.001), and mRS scores of 1 and below (*OR* = 3.291, *p* = 0.031) had a greater probability of belonging to the “high level-complexity group.” Patients with a low number of comorbid other chronic diseases and self-care of no need for dependence (*OR* = 0.116, *p* = 0.003) and mild dependence (*OR* = 0.174, *p* = 0.013) had a high probability of belonging to the “low level-unpredictable group.”

When the “medium level group” was compared with the “low level-unpredictable group,” patients with less than college education, per capita monthly household income <1,000 CNY (*OR* = 9.537, *p* = 0.001), and mRS scores of 2 (*OR* = 6.500, *p* = 0.002) had a greater probability of belonging to the “medium level group.” Patients with number of comorbid other chronic diseases at 0 and 1, self-care as no need for dependency (*OR* = 0.293, *p* = 0.041) had a higher probability of belonging to the “low level-unpredictable group.”

### 3.5 Comparison of latent classes of illness uncertainty in perceived social support scores

An analysis of variance (ANOVA) was used to explore the effect of different latent classes of illness uncertainty on perceived social support, using the total perceived social support score and the three-dimensional scores as outcome variables. The results showed statistically significant differences in total perceived social support, family support, and other support scores for the three latent classes of illness uncertainty (*p* < 0.05) (Table 4).

**Table 4:** Comparison of perceived social support scores for the three latent illness uncertainty classes.

Variables	Group			F	*P*	LSD
	Class 1 (*n* = 69)	Class 2 (*n* = 290)	Class 3 (*n* = 61)			
Perceived social support	65.12 ± 6.37	62.38 ± 5.91	61.10 ± 6.56	7.894	<0.001	C1 > C2, C1 > C3
Family support	24.52 ± 2.63	23.08 ± 2.73	22.39 ± 2.70	11.157	<0.001	C1 > C2, C1 > C3
Other support	20.10 ± 3.14	19.34 ± 2.94	18.59 ± 3.04	4.151	0.016	C1 > C2, C1 > C3
Friend support	20.49 ± 2.19	19.96 ± 1.99	20.13 ± 2.30	1.891	0.152	

Class 1, low-level-unpredictability group; Class 2, medium-level group; Class 3, high-level-complexity group.

## 4 Discussion

The present study identifies the existence of three latent classes of illness uncertainty in ischemic stroke patients based on a combination of latent class model metrics, which is similar to the results of studies in other disease latent classes (Yu et al., 2023). Three latent classes of patients exhibit different characteristics of illness uncertainty, which can help clinical practitioners identify differences in patient characteristics in order to carry out individualized care interventions. Patients in the “low level-unpredictable group” had the lowest level of illness uncertainty but the highest scores on the unpredictability dimension, and such patients may believe that ischemic stroke is characterized by a high rate of recurrence and that it is difficult to predict the progression of the disease and its prognosis. A qualitative study found that uncertainty about future events (stroke recurrence) and discrimination of stroke signs and symptoms were major sources of illness uncertainty in stroke survivors (Peng et al., 2016). Patients in the “high-level-complexity group” had high scores on the complexity dimension, which may indicate that they perceive the treatment and care process of ischemic stroke as extremely complex and difficult to fully understand. This complexity may be one of the main sources of uncertainty about the illness in this group of patients. Previous research has shown that illness uncertainty is negatively associated with disease knowledge (Liu et al., 2020). Increasing patients’ level of disease awareness can reduce the level of illness uncertainty to some extent. Healthcare professionals are one of the most important ways for patients to gain knowledge about the disease. Therefore, it is recommended that healthcare professionals popularize the knowledge of stroke disease and also pay attention to their psychological changes, conduct timely psychological assessment and implement comprehensive interventions to reduce the illness uncertainty.

The results of this study showed that patients living in rural areas and a monthly per capita household income of <1,000 CNY had a higher probability of belonging to the “high-level-complexity group.” The reason may be that living in rural areas for a long period of time, they have access to fewer medical resources and a single source of disease-related information, which makes them more susceptible to illness uncertainty. A study by Ni found that economic status is an important factor influencing the feeling of illness uncertainty in stroke patients (Ni et al., 2018). Some ischemic stroke patients, in addition to hospitalization in the acute phase, have a lengthy rehabilitation phase after discharge from the hospital, implying high medical costs for the patient. Patients with low incomes who are faced with significant financial pressures often have uncertainty about the effectiveness of their treatment and their prognosis, thus exacerbating their illness uncertainty. In contrast, patients with relatively low financial stress have better healthcare resources and coping skills, and their level of illness uncertainty is lower. Patients with a lower level of education were more likely to belong to the “medium level group.” Study finds patients’ educational level negatively correlated with illness uncertainty (Zhang et al., 2024). The reason for this may be that patients with higher educational levels can seek disease-related information by looking through books or using the Internet, and have a better ability to analyze and deal with problems and communicate with healthcare professionals (Van Heel et al., 2023), resulting in a low level of illness uncertainty. The lower the number of comorbid other chronic diseases, the higher the probability of belonging to the “low level-unpredictable group.” The reason may be that many chronic diseases, such as hypertension, diabetes, and hyperlipidemia, are antecedents to ischemic stroke (Liu et al., 2024; Sarfo et al., 2020). Complexity of treatment options for patients with multiple chronic conditions, patient uncertainty about disease outcomes, and concerns about the high recurrence rate of ischemic stroke. The mRS score reflects the patient’s functional status and prognosis (de Havenon et al., 2021). In this study, patients with mRS scores of 1 or less were more likely to belong to the “high-level-complexity group,” which is different from the findings of previous studies. The reason may be that patients with mRS scores of 1 and below have low disease severity, worry about disease recurrence after recovery, and are more willing to obtain disease-related information through multiple channels, but this information may be inaccurate or overly complex, exacerbating their concerns about their condition and leading to the presence of high levels of illness uncertainty. A qualitative study of patients said that when patients receive too much information about their illness, it does not facilitate their understanding of the circumstances surrounding the illness (Leblanc et al., 2017).

We also found that the “low level-unpredictable group” patients had higher perceived social support, which is consistent with previous research findings (Chen et al., 2022; Hurt et al., 2017). Patients in the “low level-unpredictable group” may have higher psychological resilience and are more inclined to seek external support to alleviate this uncertainty (Yang et al., 2015; Yu et al., 2024). In terms of family support, patients in the “low level-unpredictable group” were willing to initiate discussions with family members about their treatment and rehabilitation plans or to express their needs, which prompted family members to be more willing to be consistently engaged, enhancing the patients’ sense of security and feeling of being cared for. In terms of other supports, patients in the “low level-unpredictable group” may be more receptive to healthcare professionals’ advice and guidance and willing to engage in positive communication. On the contrary, patients in the “medium level group” or “high-level-complexity group” may not be conducive to communication and interaction with healthcare professionals because they feel confused and helpless about various aspects of the disease, and healthcare professionals are not able to keep abreast of the patients’ needs and problems, which may affect the provision of support.

The present study found significant heterogeneity in illness uncertainty among ischemic stroke patients through latent class analysis, and it was significantly associated with demographic characteristics and level of collateral social support. Based on this, this study provides the following specific and feasible practical recommendations for clinical care and stroke management: (1) Nursing strategies for different classes of illness uncertainty. For patients in the “low level-unpredictable group” of illness uncertainty, condition monitoring and communication can be strengthened to reduce the patient’s fear of the unknown; for patients in the “medium level group,” a comprehensive assessment can be conducted and targeted health education can be provided; and for patients in the “high-level-complexity group,” multidisciplinary collaboration, psychological interventions, and support system integration can be implemented. (2) Targeted intervention for specific groups. For patients living in rural areas, regular clinics can be conducted in rural areas to provide free health checks and rehabilitation guidance to help patients better manage their diseases; knowledge of stroke prevention and rehabilitation can be popularized through community bulletin boards and radio broadcasts to raise patients’ health awareness. For patients with a low per capita monthly family income, assistance can be provided to help them apply for medical assistance to alleviate their financial burden. For patients with a high number of comorbidities of other chronic diseases, comprehensive disease management programs are formulated for patients; knowledge on the management of multiple chronic diseases is provided to patients and their families to help them better coordinate the treatment of different diseases. (3) Nursing strategies based on a social support system. Enhance family support and expand the social support network, especially for patients in the “high-level-complexity group,” healthcare professionals can provide disease knowledge training and nursing skills training for family members, and encourage family members to participate in the patient’s disease management; and form a mutual support group to obtain more psychological support and rehabilitation experience through communication and sharing with other patients.

### 4.1 Limitations

This study has some limitations. The study population was ischemic stroke patients in the inpatient unit and did not consider patients in the community; the scope of the questionnaire collection was limited to Yibin City, China, which restricted the generalizability of the findings. This study mainly focused on the specificity of illness uncertainty dimensions, and the discussion section failed to adequately consider the combined effects of general demographic information and disease-related information of various types of patients, and will focus on these variables in subsequent studies. Convenience sampling method was used in this study, which may have some selection bias, and other more rigorous sampling methods, such as stratified sampling will be considered in subsequent studies to improve the representativeness of the sample and the extrapolation of the findings. Lastly, this study was a cross-sectional survey, which did not allow for the tracking and observation of changes in illness uncertainty in ischemic stroke patients. In the future, the sample size should be expanded, multicenter studies should be conducted, and longitudinal studies should be added to understand the dynamic changes of illness uncertainty in ischemic stroke patients.

## 5 Conclusion

The results of this study suggest that ischemic stroke patients’ illness uncertainty has obvious latent class characteristics, and each latent class of patients has different perceived social support. Healthcare professionals should individualize interventions for different latent classes of patients. Healthcare professionals should individualize interventions for different latent classes of patients to reduce illness uncertainty and improve quality of life for patients with ischemic stroke.

## Data Availability

The raw data supporting the conclusions of this article will be made available by the authors, without undue reservation.
